# Current News

**Published:** 1905-11

**Authors:** 


*Secretary.*

---

### Current News.

### XV CONGRÈS INTERNATIONAL DE MÉDECINE, LISBONNE, 19–26 AVRIL, 1906.

### REGLEMENT.

Article 1.—Le XV Congrès International de Médecine s'ouvrira à Lisbonne le 19 avril 1906 et sera clos le 26 du même mois. Son but est exclusivement scientifique.

Art. 2.—Seront membres du Congrès:

1.°—Les Médecins qui en feront la demande.

2.°—Les savants qui seront présentés par le comité exécutif portugais ou par les comités nationaux étrangers.

Art. 3.—Tout membre du Congrès recevra sa carte d'identité, après avoir fait parvenir sa cotisation au Trésorier général du Congrès. Cette carte sera nécessaire pour pouvoir profiter des avantages faits aux Congressistes. Le versement à faire est de 25 *Francs* ou 20 *Marks* ou 1 *Livre Sterling* pour les Membres étrangers; de 5$ 500 *réis* pour les membres nationaux.

Les comités étrangers peuvent recevoir les adhésions de leurs nationaux. Ils en transmettront les cotisations au Secrétariat général portugais, qui leur enverra immédiatement un nombre de cartes égal à celui des cotisations transmises.

Art. 4.—En faisant parvenir leur cotisation au trésorier, les membres du Congrès devront indiquer lisiblement leurs nom, qualités et adresse, la section où ils veulent s'inscrire, et joindre leur carte de visite.

Art. 5.—En dehors des autres avantages, chaque membre du Congrès aura droit au volume de la partie générale du Congrès et aux travaux imprimés de la section dont il fait partie.

Art. 6.—Les sections du Congrès sont les suivantes:

(1) Anatomie (anatomie descriptive et comparée, anthropologie, embryologie, histologie).

(2) Physiologie.

(3) Pathologie générale, bactériologie et anatomie pathologique.

(4) Thérapeutique et pharmacologie.

(5) Médecine.

(6) Pédiatrie.

(7) Neurologie, psychiatrie et anthropologie criminelle.

(8) Dermatologie et syphiligraphie.

(9) Chirurgie.

(10) Médecine et chirurgie des voies urinaires.

(11) Ophthalmologie.

(12) Laryngologie, rhinologie, otologie et stomatologie.

(13) Obstétrique et gynécologie.

(14) Hygiène et épidémiologie.

(15) Médecine militaire.

(16) Médecine légale.

(17) Médecine coloniale et navale.

Art. 7.—Un Comité exécutif et une Commission générale d'organisation sont chargés de la préparation et du fonctionnement du Congrès.

Art. 8.—Le Congrès tiendra séance chaque jour, soit en assemblées générales, soit en réunions de sections.

Art. 9.—Deux assemblées générales auront lieu, l'une le jour de l'ouverture, l'autre le dernier jour du Congrès.

Il y aura en outre les assemblées extraordinaires que l'on jugera nécessaires pour la discussion de sujets généraux ou pour la présentation de conférences scientifiques; leur programme sera fixé par le Comité exécutif.

Les conférences ne peuvent pas être suivies de discussion.

Art. 10.—Il sera procédé le jour de la première assemblée générale à la proclamation des présidents d'honneur du Congrès.

Art. 11.—Les assemblées générales d'ouverture et de clôture seront consacrées aux discours d'usage et aux vœux à émettre. Ne pourront prononcer de discours dans ces assemblées générales que les membres qui auront été désignés et invités par le Comité exécutif du Congrès.

Art. 12.—Toutes les propositions relatives aux travaux du

Congrès devront être notifiées au Comité exécutif avant le 1.er janvier 1906. Le Comité décidera sur la suite à donner à ces propositions.

Art. 13.—Les communications se référant aux travaux du Congrès doivent parvenir au Secrétariat général avant le 1.er janvier 1906; celui-ci se chargera de leur transmission à la section respective.[1]

Les titres des communications devront être accompagnés d'un court résumé (en forme de conclusions, si possible) ; cet extrait sera imprimé par les soins du Comité exécutif du Congrès et distribué aux membres de la section correspondante.

Art. 14.—On pourra présenter des communications après le 1.er janvier 1906 et même pendant le Congrès, mais elles ne pourront être mises à l'ordre du jour qu'après discussion de celles présentées dans le délai prescrit.

Art. 15.—Chaque Comité de section organisera son programme de travail (audition des rapports et discussion des sujets proposés, communications diverses).

Art. 16.—Deux ou plusieurs sections peuvent se réunir en une seule assemblée pour des travaux en commun.

Art. 17.—Les membres du Congrès peuvent prendre part aux travaux des sections où ils ne se sont pas fait inscrire.

Art. 18.—Les discours prononcés en assemblée générale et les rapports faits dans les sections seront publiés dans les comptes rendus des travaux Congrès; pour les communications diverses et discussions, le Comité exécutif se réserve tout droit d'examen. Le temps assigné à chaque communication ne pourra pas dépasser quinze minutes, et les orateurs qui prendront part à la discussion ne pourront parler plus de cinq minutes chacun.

Les auteurs des rapports et communications auront dix minutes pour leur réponse générale.

Art. 19.—Le texte écrit des rapports, communications et discussions, devra être remis le jour même au Secrétaire de la section respective. De même pour les discours en assemblée générale à remettre au Secrétaire général.

---

[1] Les *communications libres* ne doivent par être confondues avec les *rapports officiels.* Pour ceux-ci, le Comité exécutif a fixé le 30 septembre 1905 pour la présentation au Secrétariat général, a fin qu'ils puissent être imprimés avant l'ouverture du Congrès (*Note du Secrétaire général*).

Art. 20.—La langue française est la langue officielle du Congrès pour les relations internationales. Dans les assemblées générales ainsi que dans les sections, les langues allemande, anglaise et française pourront être employées.

Dans les sections on pourra faire usage d'une autre langue, pourvu qu'un des membres présents en fasse la traduction immédiate dans une des langues permises.

Art. 21.—Toutes les questions ayant trait aux travaux scientifiques des sections doivent être soumises et adressées au Président du Comité de la section intéressée. Pour tout ce qui concerne l'organisation et le fonctionnement du Congrès, on devra s'adresser au Secrétaire général.

Art. 22.—Dans sa dernière assemblée générale, le Congrès désignera le siège de sa prochaine réunion et en elira le bureau.

Art. 23.—Les dames des congressistes auront leur entrée au Congrès dans des conditions qu'on réglera, moyennant le versement d'une demie cotisation.

*Comité exécutif.*—Président, Cons. Costa Alemao; Secrétaire général, Prof. Miguel Bombarda (Hôpital de Rilhafolles, Lisbonne); Trésorier, Alfredo Luiz Lopes; Secrétaires, Antonio de Azevedo, Mello Breyner, Azevedo Neves, Mattos Chaves (Fernando); Membres, Annibal Bettencourt, Prof. Clemente Pinto, Prof. Daniel de Mattos, Prof. Ricardo Jorge, Silva Carvalho, Zeferino Falcao.

SECTIONS.

1.re—Président, José Antonio Serrano, Lisbonne; Secrétaire resp., Marck Athias, Rua de Santa Martha, 144, Lisbonne.

2.me—Président, Philomeno da Camara, Coimbra; Secrétaire resp., Arthur Cardoso Pereira, Rua Conselheiro Pedro Franco, 42, Lisbonne.

3.me—Président, Pedro Bettencourt Raposo, Lisbonne; Secrétaire resp., Annibal Bettencourt, Real Instituto Bacteriologico, Lisbonne.

4.me—Président, Raymundo Motta, Coimbra; Secrétaire resp., Brito Camacho, Hôpital de Rhilhafolles, Lisbonne.

5.me—Président, Bettencourt Pitta, Lisbonne; Secrétaire resp., Benjamin Arrobas, Campo dos Martyres da Patria, 28, Lisbonne.

6.me—Président, Dias d'Almeida, Porto; Secrétaire resp., Jayme Salazar De Sousa, Avenida Fontes Pereira de Mello, D, Lisbonne.

7.me—Président, Caetano Beirao, Lisbonne; Secrétaire resp., Virgilio Machado, Avenida da Liberdade, 200, Lisbonne.

8.me—Président, Zeferino Falcao, Lisbonne; Secrétaire resp., Mello Breyner, Rua da Junqueira, 59, Lisbonne.

9.me—Président, Oliveira Feijao, Lisbonne; Secrétaire resp., Augusto de Vasconcellos, Rua Nova do Almada, 80, Lisbonne.

10.me—Président, Moraes Caldas, Porto; Secrétaire resp., Arthur Furtado, Rua de S. Roque, 100, Lisbonne.

11.me—Président, Sousa Refoios, Coimbra; Secrétaire resp., —— ——.

12.me—Président, Gregorio Fernandes, Lisbonne; Secrétaire resp., Avelino Monteiro, Avenida da Liberdade, 91, Lisbonne.

13.me—Président, Candido de Pinho, Foz, Porto; Secrétaire resp., Daniel de Mattos, Hôpital de Rilhafolles, Lisbonne.

14.me—Président, Ricardo Jorge, Lisbonne; Secrétaire resp., Guilherme Ennes, Rua do Livramento, 50, Lisbonne.

15.me—Président, Cunha Bellem, Lisbonne; Secrétaire resp., Manoel Giao, Avenida da Liberdade, 115, Lisbonne.

16.me—Président, Silva Amado, Lisbonne; Secrétaire resp., Lima Duque, Calçada da Estrella, 131, Lisbonne.

17.me—Président, Cons. Ramada Curto, Lisbonne; Secrétaire resp., Silva Telles, Rua Saraiva de Carvalho, 14, Lisbonne.

---

### FIRST ANNUAL CLINIC OF THE FRATERNAL DENTAL SOCIETY OF ST. LOUIS.

Special features of the meeting to be held November 20 and 21, at the Barnes Dental College, will be a series of lectures on "Cavity Preparation," "Methods and Principles of packing Gold," and "Methods and Principles of finishing Fillings," by Dr. E. K. Wedelstaedt of St. Paul.

The following well-known members of the Black and Wedelstaedt Clubs will be present and clinically demonstrate "extension for prevention" to its fullest extent: Drs. A. C. Searle, Owatonna, Minn., J. F. Wallace, Canton, Mo., C. W. Booth, Cedar Rapids, Iowa, J. J. Booth, Marion, Iowa, William Finn, Cedar Rapids, Iowa, J. B. Pherrin, Central City, Iowa, Ed. S. Brown, Edina,

Mo., W. T. Rutledge, Monroe City, Iowa, and S. E. Wallace, La Belle, Mo.

Porcelain work will be fully demonstrated by Drs. F. E. Roach, Chicago, W. L. Ellerbeck, Salt Lake City, Geo. T. Banzett, Chicago, W. H. Cudworth, Milwaukee, and Craig W. Work, Ottumwa, Iowa.

Other clinics, on various subjects, will be given by Drs. W. L. Reed, Mexico, Mo., J. B. Howell, Paducah, Ky., C. L. Rose, Fargo, N. D., F. B. Lawrence, Eldorado, Kan., Geo. D. Sitherwood, Bloomington, Ill., A. Gaiser, Davenport, Iowa, Fred. Westerfield, St. Charles, Mo., Otto J. Fruth, St. Louis, Richard Summa, St. Louis, and others.

The following dealers have signified their intention to be present and display their exhibits: S. S. White Dental Mfg. Co., Dr. Jenkins Porcelain, Klewe & Co., A. C. Clark & Co., St. Louis Dental Mfg. Co., John Nolde Dental Mfg. Co., Risey Dental Mfg. Co., Denthol Chemical Co., Lambert Pharmacal Co., Lee S. Smith & Sons, Century Dental Laboratory Co., W. M. Berry Dental Laboratory Co., Sanitol Chemical Co., R. C. Brophy & Co., Keeton Williams Gold Co., Horlicks Food Co., Kress & Owens, Oakland Chemical Co., McKesson & Robbins, and others.

The Western Passenger Association and South Western Excursion Bureau have granted a rate of one and one-third fare, plus twenty-five cents validation fee, certificate plan, for this meeting for the States of Missouri, Iowa, Minnesota, Kansas, Nebraska, and Illinois, on and west of the line of the Chicago and East Illinois Railroad.

Headquarters will be at the Jefferson Hotel, Twelfth and Locust Streets. Rooms for one, without bath, $1.50 and up; rooms with bath, $2.50 and up. Rooms for two, without bath, $2.00 and up; rooms with bath, $3.00 and up.

Exhibit space may be obtained by application to the Secretary. If you have a clinic to give, send your name at once to the Supervisor of Clinics.

A cordial invitation is extended to the profession to be present and assist in making this meeting, limited in scope but limitless in importance, the best ever held in this section.

D. O. M. LeCron, *Supervisor of Clinics.*

S. H. Voyles, *Secretary.*

Burton Lee Thorpe, *President.*

